# Grouping behaviour impacts on the parasitic pressure and squamation of sharks

**DOI:** 10.1098/rspb.2022.0093

**Published:** 2022-05-25

**Authors:** Humberto G. Ferrón, Jose F. Palacios-Abella

**Affiliations:** ^1^ Cavanilles Institute for Biodiversity and Evolutionary Biology, University of Valencia, Calle Catedratico Jose Beltran Martinez 2, Paterna 46980, Spain; ^2^ School of Earth Sciences, University of Bristol, Life Sciences Building, Tyndall Avenue, Bristol BS8 1TQ, UK

**Keywords:** grouping behaviour, sharks, ectoparasitic pressure, squamation, phylogenetic comparative methods

## Abstract

The evolution of grouping behaviour involves a complex trade-off of benefits and costs. Among the latter, an increase in the risk of parasitic transmission is a well-documented phenomenon that has likely promoted the evolution of defensive mechanisms in aquatic vertebrates. Here, we explore the relationship between grouping behaviour, parasitic richness (∼parasitic pressure), and the evolution of potential defensive traits in the squamation of sharks through phylogenetic, standard and zero-inflation regression models. Our results demonstrate that sharks that frequently aggregate show increased parasitic pressure, which may constitute an agent of selection. Accordingly, their squamation is characterized by large-scale crown insertion angles and low-scale coverage, which are interpreted as traits that compromise parasite attachment and survival. These traits are less evident in regions of the body and ecological groups that are subjected to high abrasive stress or increased drag. Thus, the squamation of sharks responds to a compromise between various functions, where protective and hydrodynamic roles prevail over the rest (e.g. ectoparasitic defence and bioluminescence aiding). This work establishes a quantitative framework for inferring parasitic pressure and social interaction from squamation traits and provides an empirical basis from which to explore these phenomena through early vertebrate and chondrichthyan evolution.

## 1. Introduction

Grouping behaviour is a widespread phenomenon among aquatic vertebrates [1–3] that involves a complex trade-off of benefits and costs associated with intraspecific and interspecific interactions [4]. Possible drivers for the evolution of grouping behaviour in aquatic vertebrates include advantages on swimming energetics and migration [5], reduced vulnerability to predators due to dilution or confusion effects [6,7], and cooperative hunting [8], among others. However, the establishment of groups may also entail a series of disadvantages, such as a decrease in foraging efficiency [9] or an increased risk of parasitism [10–13]. The latter is particularly relevant in the case of monoxenic parasites (i.e. those with direct life cycles), which require only one host to complete their development and, therefore, the probability of successful transmission is enhanced by higher host densities [14].

Parasitic interactions are an essential component of aquatic communities [15,16]. They trigger the establishment of evolutionary ‘arms races’ between hosts and parasites and promote the evolution of defensive adaptations in the former [17], which may include physiological and immunological responses [18], behavioural strategies [10,19,20] and morphofunctional traits [21]. Most monoxenic parasites of primarily aquatic vertebrates are ectoparasites that predominantly anchor to the branchial or skin surfaces (i.e. Monogenea, Copepoda, Hirudinea and Thecostraca) [22,23]. These groups cause significant damage to the respiratory organs and epidermal structures, resulting in the exposure of internal tissues and facilitating the development of infections [22–24]. Hence, several authors have suggested that the squamation of sharks plays a primary role in preventing the settlement of ectoparasites [25,26].

The squamation of sharks is a complex anatomical structure consisting of minute dermal denticles (i.e. placoid scales) that display topological, ontogenetic, sexual and interspecific morphological variation highly correlated with different scale functions and ecological attributes. Consequently, a well-defined categorization of placoid scales into morphotypes and functional types has been established in the literature [21,26–28] (electronic supplementary material, figure S1): the gracile carinated scales present in active pelagic species are involved in drag reduction [29–31]; the robust densely packed scales with a rhomboidal or rounded shape that are common in all sharks in areas of the body that are subjected to high abrasive stress (i.e. the rostrum, mouth and leading edges of the fins), and covering most of the body in demersal sharks, are associated with protection against abrasion or stabilization of the leading edges of the fins [32]; the star-shaped scales with concave facets and needle-like scales accommodate photophores and permit the passage of the light emitted by those organs in bioluminescent species [33,34]; the hook- or thorn-like scales found on the skin of slow schooling sharks are presumed to play a role in preventing the settlement of ectoparasites and epibionts [21,26,27]; and the strongly carinated scales with well-developed lateral wings that cover the bodies of slow-moving species in open water, and the dorsolateral region of demersal species that live on soft substrates, are associated with more generalized functions [27] (see also [35,36] for more specific roles of placoid scales). Although hydrodynamic, abrasion resistance and bioluminescence-aiding functions are well proven in previous experimental and/or observational studies [26,33,37–41], empirical evidence to support a potential defensive role against ectoparasites is anecdotical [42–44] or even inconsistent ([21] and references therein).

Resolving the interrelations regarding grouping behaviour, (ecto)parasitic pressure and potential defensive morphological adaptations in the squamation of sharks is, therefore, critical to (1) achieve an improved understanding of the trade-offs and costs associated with the evolution of social behaviours and the drivers of parasitic interactions in sharks and, more generally, in aquatic vertebrates and (2) uncover potential morphofunctional signals in the squamation of sharks associated with grouping behaviour and the inherent risk of increased parasitic pressure, thus, providing a framework to indirectly infer and track these phenomena through chondrichthyan and early vertebrate evolution. Here, through the implementation phylogenetic, standard and zero-inflated regression models, we explore whether grouping behaviour in sharks is associated with higher parasitic pressure and assess whether this can be linked to specific squamation traits that may prevent the settlement and compromise the survival of ectoparasites, with direct implications in the design of antifouling biomimetic surfaces.

## 2. Material and methods

### (a) Database compilation

We generated a database on 213 shark species that included information on parasitic richness (PR) (i.e. the number of parasitic species in a given host species), the occurrence of grouping behaviour, and ecology (electronic supplementary material, table S1 and figures S2 and S3). Parasitic richness was calculated for each species based on records compiled by Pollerspöck & Straube [45], and only the main groups of monoxenic ectoparasites of sharks were considered (i.e. Monogenea, Copepoda, Hirudinea and Thecostraca). The shark species were categorized according to the frequency they present grouping behaviour, based on the information provided by Ebert *et al*. [46], the IUCN [47] and Froese & Pauly [48]. We classified the species into ‘taxa that show grouping behaviour infrequently’ (i.e. always found as solitary individuals or with sporadic records of aggregations) and ‘taxa that show grouping behaviour frequently’ (mostly recorded in aggregations) (GB1). In order to test the sensitivity of the model outcomes to uncertainty in this classification, we considered a second categorization in which the former category was split into ‘solitary taxa’ and ‘taxa that show grouping behaviour occasionally’ (GB2). The occurrence of grouping behaviour was considered to be unknown for species with poorly studied ethology and ecology (according to [46]), which were excluded from subsequent analyses. The shark species were further categorized into ecological groups (EG) following the classification system proposed by Ferrón & Botella [27] (i.e. strong-swimming pelagic species, schooling species of low to moderate speed, demersal species on rocky substrates and in caves, demersal species on sandy and muddy substrates, mesopelagic bioluminescent species, and slow species of the open water) and based on the ecological data reported by Ebert *et al*. [46], the IUCN [47], and Froese & Pauly [48]. Finally, we also recorded the number of published studies (NS) concerning each of the considered shark species based on the bibliographic references listed in Pollerspöck & Straube [45].

### (b) Shark squamation

**Figure 1.**
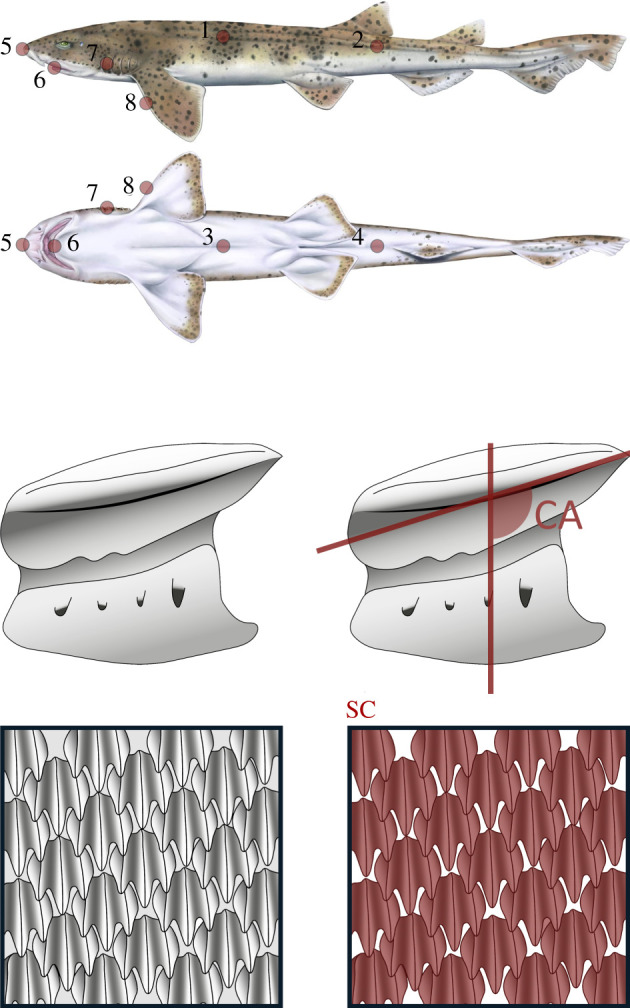
Topological positions of the placoid scale samples (upper), and the measurements considered in this study (lower). Topological positions: 1, dorsolateral region posterior to the pectoral girdle; 2, dorsolateral region posterior to the pelvic girdle; 3, ventral region posterior to the pectoral girdle; 4, ventral region posterior to the pelvic girdle; 5, tip of the rostrum; 6, most anterior part of the lower jaw; 7, gill slit between the first and second gills; 8, leading edge of the pectoral fin. Samples 1, 2, 7 and 8 were taken from the left side of the body. Measurements: CA, scale crown insertion angle; SC, scale coverage. The scale in the upper panel is presented in lateral view. The shark drawings are courtesy of Hugo Salais (HS Scientific Illustration). (Online version in colour.)

Simultaneously, we studied the squamation of 63 shark specimens of 49 different species from the collections of the Museum für Naturkunde of Berlin (MfN, Germany) and the Australian Museum of Sydney (AM, Australia) (electronic supplementary material, table S2). This selection represented suitable coverage of the diversity of the lifestyles displayed by sharks and included representatives of all the ecological groups described by Ferrón & Botella [27]. We acquired skin samples from eight different topological positions (P) (figure 1) using dermatological punches that had a diameter of 5 mm and 10 mm. The samples were cleaned mechanically (using soft toothbrushes and distilled water at high pressure) and chemically (using sodium hypochlorite [NaOCl, 5–6.5%]), according to the procedure described by Reif [26]. The samples were then visualized under a Leica Z16/DFC500 macroscope and analysed using ImageJ software [49] at the Cavanilles Institute for Biodiversity and Evolutionary Biology at the University of Valencia (Spain). For each sample, we measured the scale crown insertion angle (CA) (the angle of the crown of the scale to the vertical axis), which was averaged from measurements taken of ten scales that were randomly selected from each sample, and the scale coverage (SC) (the percentage of the skin surface covered by scales), which was measured in an area of 2 × 2 mm located at the centre of each sample (figure 1). These two variables were considered as they were suggested by several authors as potential traits that affect the attachment and survival of ectoparasites [21,44] (see §4 for further details).

### (c) Phylogenetic comparative methods

The relationship between grouping behaviour, parasitic pressure and squamation traits was assessed in two successive steps, by implementing phylogenetic generalized least squares (pGLS) models in the package ‘caper’ v. 1.0.1 [50] using R [51]. This methodology allows for the fitting of a linear regression to explore the impact of one or several predictor variables on a single response variable while controlling for a potential phylogenetic signal in the response [52]. First, we tested whether parasitic pressure was increased by the frequency of grouping behaviour by performing pGLS regressions between the PR and all possible combinations of the predictors GB1/GB2 and NS. PR was considered to be an approximation of parasitic pressure according to previous studies ([53] and references therein). The NS was incorporated to account for a potential bias of PR due to uneven sampling effort [54]. Due to the possibility that some of the records where PR is equal to zero represent true absence of parasites (i.e. structural zeroes), we checked for zero-inflation in the models using the R package ‘performace‘ v. 0.8.0 [55] and ran zero-inflated count data regressions using the R package ‘pscl’ v. 1.5.5 [56]. Second, we assessed whether the CA and SC varied according to the location on the body, lifestyle and/or frequency of grouping behaviour by performing a second set of pGLS regressions between CA/SC and all possible combinations of the predictors P, EG and GB1/GB2.

In all cases, we included null models (i.e. without covariates) for comparison. The Akaike information criterion (AIC) and likelihood-ratio tests for non-nested models (LRT) were employed to compare the goodness of fit for the different models. LRT were performed using the R package ‘nonnest2’ v. 0.5.5 [57] based on Vuong's [58] theory. Model parameters were standardized using the R package ‘effectsize’ v. 0.6.0.1 [59]. Multicollinearity was checked by calculating variance-inflation factors (VIF) and generalized variance-inflation factors (GVIF) with the R package ‘car’ v. 3.0.12 [60] and considering a threshold of GVIF^(1/(2*Df))^ = 2 (equivalent to VIF = 4) [61]. All pGLS analyses were conducted with consideration of the phylogenetic tree provided by Vélez-Zuazo & Agnarsson [62] after trimming the taxa that were not included in the respective datasets using the R package ‘ape’ v. 5.5 [63]. Species for which the squamation of more than one specimen was studied were included as polytomies that were resolved randomly with zero branch length. Additionally, all the analyses were repeated using non-phylogenetic generalized least square models (GLS).

## 3. Results

Parasitic richness varies considerably among species with different frequencies of grouping behaviour and numbers of published studies (electronic supplementary material, figure S3). The best-supported pGLS model (PR ∼ NS + GB1) indicates that parasitic richness is mostly explained by these two predictors (electronic supplementary material, table S3). This model supports that the taxa that show infrequent grouping behaviour have lower PR than those that show frequent grouping behaviour and that PR increases with the number of published works (table 1). This is also supported by the models that include a finer categorization of grouping behaviour (i.e. PR ∼ NS + GB2), in which the PR increases successively in solitary species, species that show occasional grouping behaviour, and species that show frequent grouping behaviour (electronic supplementary material, data S1). Grouping behaviour affects PR even in models that do not account for the number of published studies, but these explain a lower amount of variation and have high AIC scores (electronic supplementary material, table S3 and data S1). Two additional candidate models (i.e. PR ∼ NS + GB2; PR ∼ NS × GB1) show a similar support on the basis of the AIC scores (i.e. ΔAIC < 2) and LRT results (*p*-value = 0.73 and 0.64, respectively) (electronic supplementary material, table S3).

**Table 1.** Details of the best-supported phylogenetic (pGLS) and standard (GLS) regression models, showing the associated coefficients and *p*-values. CA, scale crown insertion angle; PR, parasitic richness; SC, scale coverage. Predictors: EG, ecological group (School, schooling species of low to moderate speed; Biolu, mesopelagic bioluminescent species; DemRo, demersal species on rocky substrates and in caves; DemSa, demersal species on sandy and muddy substrates; Pelag, strong-swimming pelagic species; Open, slow species of the open water); GB1, grouping behaviour (Infrequent, Frequent); GB2, grouping behaviour (Solitary, Occasional, Frequent); NS, number of studies; P, position (the numbers refer to the topological positions in figure 2).

	pGLS						LM					
	PR ∼ NS + GB1		CA ∼ P + EG + GB1		SC ∼ P + EG + GB2		PR ∼ NS + GB1		CA ∼ P + EG + GB1		SC ∼ P + EG + GB2	
	estimate	*p*-value	estimate	*p*-value	estimate	*p*-value	estimate	*p*-value	estimate	*p*-value	estimate	*p*-value
intercept	0.54	6.51 × 10^−1^	134.65	2.20 × 10^−16^	41.36	5.35 × 10^−13^	1.81	4.39 × 10^−2^	129.71	2.00 × 10^−16^	50.01	2.00 × 10^−16^
NS	0.02	2.00 × 10^−16^	—	—	—	—	0.02	2.00 × 10^−16^	—	—	—	—
GB1	—	—										
frequent	0.00	—	—	—	0.00	—	0.00	—	—	—	0.00	—
infrequent	−2.81	1.23 × 10^−2^	—	—	6.34	1.36 × 10^−1^	−3.23	8.55 × 10^−4^	—	—	4.65	6.21 × 10^−2^
GB2												
frequent	—	—	0.00	—	—	—	—	—	0.00	—	—	—
ocassional	—	—	−2.80	5.80 × 10^−1^	—	—	—	—	−2.59	3.42 × 10^−1^	—	—
solitary	—	—	−15.46	2.80 × 10^−2^	—	—	—	—	−12.65	1.07 × 10^−6^	—	—
P												
P1	—	—	0.00	—	0.00	—	—	—	0.00	—	0.00	—
P2	—	—	0.07	9.77 × 10^−1^	1.26	7.56 × 10^−1^	—	—	−0.08	9.80 × 10^−1^	0.90	8.06 × 10^−1^
P3	—	—	−0.98	6.99 × 10^−1^	1.84	6.49 × 10^−1^	—	—	−1.87	5.52 × 10^−1^	1.81	6.22 × 10^−1^
P4	—	—	−0.13	9.58 × 10^−1^	1.36	7.36 × 10^−1^	—	—	−1.00	7.51 × 10^−1^	2.15	5.58 × 10^−1^
P5	—	—	−22.92	2.20 × 10^−16^	31.46	1.78 × 10^−13^	—	—	−17.97	2.62 × 10^−8^	22.39	3.02 × 10^−9^
P6	—	—	−11.25	1.31 × 10^−5^	32.20	5.46 × 10^−14^	—	—	−11.10	4.78 × 10^−3^	22.12	4.58 × 10^−9^
P7	—	—	−0.97	7.02 × 10^−1^	26.49	3.05 × 10^−10^	—	—	−1.21	7.02 × 10^−1^	18.97	4.06 × 10^−7^
P8	—	—	−19.75	1.59 × 10^−13^	29.34	4.73 × 10^−12^	—	—	−16.13	5.21 × 10^−7^	21.33	1.48 × 10^−8^
EG												
School	—	—	0.00	—	0.00	—	—	—	0.00	—	0.00	—
Biolu	—	—	13.94	1.32 × 10^−1^	−27.25	1.55 × 10^−5^	—	—	4.76	1.65 × 10^−1^	−28.52	6.25 × 10^−13^
DemRo	—	—	−17.42	1.32 × 10^−1^	36.94	3.70 × 10^−6^	—	—	−18.07	5.17 × 10^−11^	32.70	2.00 × 10^−16^
DemSa	—	—	−26.70	6.72 × 10^−3^	39.44	2.29 × 10^−5^	—	—	−18.99	1.76 × 10^−8^	31.64	2.31 × 10^−15^
Pelag	—	—	−20.86	1.96 × 10^−2^	35.39	5.78 × 10^−7^	—	—	−21.62	5.22 × 10^−15^	33.25	2.00 × 10^−16^
Open	—	—	−19.60	2.10 × 10^−1^	35.11	4.54 × 10^−4^	—	—	−15.91	1.38 × 10^−5^	33.02	2.91 × 10^−14^

Zero-inflated regressions also support that parasitic richness increases with the frequency of grouping behaviour and the number of published studies, according to the best-supported model (PR ∼ NS + GB2) (count model in electronic supplementary material, tables S4 and S5). No other candidate model provides a similar fit to the data on the basis of the AIC scores and LRT results. Zero-inflation model supports that the odds that a shark species has a parasitic richness of zero significantly increases in solitary species and decreases with the number of published studies (by 5.20 and 0.99, respectively; zero-inflation model in electronic supplementary material, table S5 and data S1).

**Figure 2.**
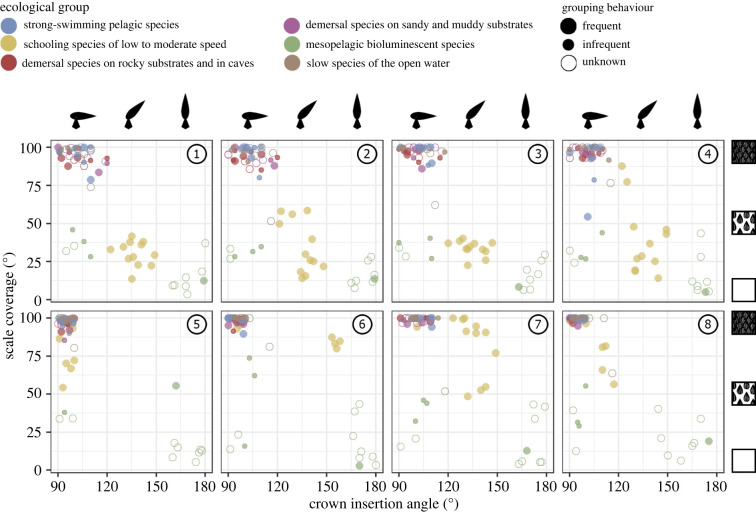
Scatterplots representing scale crown insertion angle and scale coverage in various topological positions of the studied shark specimens. The numbers refer to the topological positions in figure 2 and the specimens are coloured by ecological group. (Online version in colour.)

The scale crown insertion angle and the scale coverage vary significantly among the different species and body locations (figure 2). The scale crown insertion angle is largely explained by grouping behaviour, the topological position on the body and the ecological group, according to the best-supported pGLS model (CA ∼ P + EG + GB2) (electronic supplementary material, table S3). This model supports that the CA increases successively in solitary species, species that show occasional grouping behaviour and species that show frequent grouping behaviour. A similar CA is observed among the scales on the gill slits and dorsolateral and ventral regions of the body, while smaller CA is observed in the scales of the mouth, rostrum and leading edge of the pectoral fins (table 1 and figure 2). Schooling species of low to moderate speed display larger CA than strong-swimming pelagic species, demersal species on rocky substrates and in caves, demersal species on sandy and muddy substrates, and slow species of the open water. However, they display smaller CA than mesopelagic bioluminescent species (table 1 and figure 2). Four additional candidate models (i.e. CA ∼ P + EG; CA ∼ P; CA ∼ P + GB2; CA ∼ P + EG + GB1) show similar AIC scores but LRTs support that the model with the lowest AIC score is significantly different from all of them (*p*-value = 0.09, 0.03, 0.04 and 0.06, respectively; electronic supplementary material, table S3).

Similarly, the scale coverage is largely explained by grouping behaviour, the topological position on the body and the ecological group, according to the best-supported pGLS model (SC ∼ P + EG + GB1) (electronic supplementary material, table S3). This model supports that species that show occasional grouping behaviour have greater SC compared with species that show frequent grouping behaviour. Similar SC is observed on the dorsolateral and ventral regions of the body, while a greater SC is observed on the gill slits, mouth, rostrum and leading edge of the pectoral fins (table 1 and figure 2). Schooling species of low to moderate speed display less SC than strong-swimming pelagic species, demersal species on rocky substrates and in caves, demersal species on sandy and muddy substrates, and slow species of the open water. However, they show a greater SC compared to mesopelagic bioluminescent species (table 1 and figure 2). Two additional candidate models (i.e. SC ∼ P + EG; SC ∼ P + EG + GB2) show a similar support on the basis of the AIC scores but the *p*-value from the LRT is comparatively low for the model excluding grouping behaviour (*p*-value = 0.11; electronic supplementary material, table S3).

In terms of model support, the predictors included in the best-supported models and the significance of the factor levels, the results derived from the GLS models are virtually the same as those obtained from the pGLS models (tables 1 and S3). Candidate GLS models excluding grouping behaviour clearly fit the data less well than those including this covariate, especially when considering the results from LRTs (electronic supplementary material, table S3). We detect no significant multicollinearity among the explanatory variables (electronic supplementary material, data S1).

## 4. Discussion

### (a) Grouping behaviour involves increased parasitic pressure in sharks

The results derived from the pGLS, GLS and zero-inflated regression models demonstrate that grouping behaviour affects the richness of monoxenic ectoparasites, even when various categorizations of the former are considered (electronic supplementary material, tables S1 and S4). Species that show frequent grouping behaviour have greater parasitic richness than those that display a solitary lifestyle or aggregate occasionally (table 1 and electronic supplementary material, table S5). This agrees with the fact that solitary species might be contributing to zero-inflation (electronic supplementary material, tables S4 and S5). Parasitic richness is a reliable estimate of parasitic pressure and the impact on hosts, as shown by independent evolutionary, genetic, immunological, physiological and behavioural evidence ([53] and references therein). According to this, our study supports that shark taxa that tend to aggregate more frequently are exposed to a stronger parasitic pressure, which might constitute a selective agent for the evolution of defensive mechanisms.

Besides parasitic richness, other metrics such as intensity (i.e. average number of individual parasites of a given type present in an infected host) or prevalence (i.e. percentage of the host population infected with a parasite) are commonly used in studies that aim to quantify ecological and evolutionary consequences of parasitism [64]. The integration of those parameters within the framework here presented could provide novel insights about their interrelationships with parasitic richness and pressure [65]. However, the design of such large-scale comparative studies is hindered by the fact that intensity and prevalence records in sharks are still scarce. Other variables of potential interest, which are equally scant in the literature, include the number and density of individuals in the aggregations or information on group composition (e.g. number of species, age or sex) [1].

The results of this study also show that parasitic richness is correlated with the number of studies published on each shark species, with the PR being greater for the taxa that were studied extensively (those that included a broader bibliography). This indicates a requirement for the incorporation of variables that account for potential bias due to uneven sampling effort when studying and modelling parasitic richness rather than solely considering raw counts of parasite species [54].

### (b) Parasite control mechanisms of shark squamation

The pGLS and GLS models reveal that the scale crown insertion angle and the scale coverage are affected by the frequency of grouping behaviour (electronic supplementary material, table S3). Thus, the taxa that frequently aggregate have squamation with a larger CA and less SC than those that are mostly solitary or aggregate occasionally (table 1). We propose that this phenomenon may be a defensive response for the prevention of parasite attachment and to compromise the survival of parasites on the skin of species with increased parasitic pressure associated with grouping behaviour.

In support of this hypothesis, previous reports on ectoparasite attachment locations indicated that there is a preference for certain areas that are characterized by the absence of denticles and/or extensive wear [66]. This seems particularly true for species that have scales with crowns that point upwards, for which parasites are mostly restricted to the mouth cavity, gills and spiracle (e.g. [67–71]). Conversely, research has shown that a dense coverage of scales eases the attachment and migration of the larvae of the main groups of shark ectoparasites [44]. This is also evident in the general occurrence of copepods on the body surface of pelagic sharks that have imbricated tilted denticles (e.g. [67,72,73]). This type of squamation may further provide a suitable microhabitat for the larvae of certain parasitic groups (e.g. Monogenea and Copepoda) that often occupy the free spaces between the lower surface of the crown and the skin [74,75], while a sparse coverage of scales may increase exposure to a more hostile external environment and predators [76]. Therefore, a combination of a large CA and low SC could reduce the effective surface area for the adhesion and migration of ectoparasites and provide an inadequate microhabitat for the larvae.

### (c) The squamation of sharks is shaped by a complex trade-off of functional drivers

Besides the frequency of grouping behaviour, the pGLS and GLS models show that the scale crown insertion angle and the scale coverage also vary between the various regions of the body and the lifestyle of each species (electronic supplementary material, table S3). The topological and ecological variation of squamation has been illustrated in several previous studies ([26,27] and references therein). Here, we demonstrate that the rostrum, mouth and leading edge of the pectoral fins have scales with a smaller CA and greater SC than the dorsolateral and ventral regions of the body. Schooling species of low to moderate speed show a larger CA and less SC than the strong-swimming pelagic species, both groups of demersal species, and slow species of the open water. However, they showed a smaller CA and greater SC than the mesopelagic bioluminescent species (table 1 and figure 2).

We propose that this complex variation of patterns regarding shark squamation may have arisen due to an adaptive trade-off [32,77]. When subjected to various selective pressures (i.e. high abrasion, increased drag, increased ectoparasitism, or the presence of photophores), the optimization of the CA and SC seems to follow divergent trajectories. Thus, while a large CA and less SC may provide defence against ectoparasites (see above), a small CA and greater SC may enable abrasion resistance and drag reduction [32]. Our results suggest that morphofunctional adaptations to abrasion resistance and drag reduction prevail over those associated with defence against ectoparasites when increased abrasion or drag occurs. Accordingly, the areas of the body that are subjected to high abrasive stress (i.e. the mouth, rostrum and leading edges of the pectoral fins in all sharks, and most of the body of demersal species) and increased flow velocity (i.e. most of the body of strong-swimming pelagic species) have scales with comparatively small CA and greater SC, despite the fact that many of these pelagic and demersal species often occur in aggregations (figure 2 and electronic supplementary material, table S1) [46–48]. This view is further reinforced by the differences in the standardized coefficient magnitudes of the models, which indicate that these particular lifestyles and body regions exert a stronger effect on CA and SC than the fact of belonging to a species with a high frequency of grouping behaviour (electronic supplementary material, data S1). The scales of these body regions and ecological groups present conserved morphologies, generally as a result of convergent evolution, and are associated with the abrasion-resistant and drag-reduction functional types described by Ferrón & Botella [27].

Conversely, the squamation of bioluminescent sharks is characterized by the sparse presence of scales, enabling the accommodation of photophores without intercepting the light emitted by them [26,33,78,79] (but see also [80]). Our results support this and show that the bioluminescent species have the lowest SC among all the sharks studied (table 1 and figure 2). By contrast to species in other ecological groups, bioluminescent species show significant diversity regarding scale crown morphology (electronic supplementary material, figure S1) [26,27,33]. This fact is reflected in our results in the wide range of CA values observed among sharks of this ecological group (figure 2), suggesting that this trait plays a minor role in bioluminescence aiding. Considering this, no trade-off is expected between the morphofunctional adaptations of squamation associated with bioluminescence aiding and defence against ectoparasites when photophores and high ectoparasitic pressure co-occur in the same species. Our results show that the largest and smallest CA correspond to scales of etmopterid and dalatiid taxa (electronic supplementary material, table S2), which are mostly regarded as schooling and solitary sharks, respectively [26,33]. This indicates that part of the morphological diversity of bioluminescent shark scales could be associated with the frequency of grouping behaviour and the associated increased parasitic pressure. However, this should be cautiously considered as the ecology and ethology of these deep-water sharks remain poorly understood.

### (d) Concluding remarks and further implications

Here, we constrain the relationship between grouping behaviour, ectoparasitic pressure and associated defensive mechanisms regarding squamation in sharks through phylogenetic, standard and zero-inflated regression models. We show that a higher frequency of grouping behaviour is associated with increased parasitic pressure, which may trigger the evolution of squamation with a large CA and low SC. These are interpreted as traits that prevent the settlement and compromise the survival of ectoparasites. This phenomenon is less evident in the body regions and ecological groups that experience high abrasive stress or increased drag, suggesting that the squamation of sharks responds to a complex trade-off of functions where protective and hydrodynamic roles prevail over the rest (e.g. ectoparasitic defence and bioluminescence aiding). The analysis of these data in the context of Pareto optimality theory might provide a quantitative framework for explicitly testing this hypothesis [81,82].

The results of our study shed light on the cost–benefit balance that determines the evolution of grouping behaviour in vertebrates. Moreover, we have detected and quantified several squamation traits associated with high ectoparasitic pressure. These results constitute a basis to indirectly infer this phenomenon, not only in living taxa but also in extinct groups and may also enable us to trace the evolution of parasitic and social interactions in the evolutionary history of vertebrates and chondrichthyans in the future. The comparatively good representation of vertebrate squamations in the fossil record ensures a wide applicability of this approach to extinct taxa. Data extracted from this novel methodological framework might be crucial to complement the records of host–parasite body fossils in association [83–89] and pathologies [90,91]. Still, this more anecdotal evidence has the unique potential to provide estimations of parasitic intensity or prevalence as well as information on the nature of ectoparasites and their attachment site preferences [92–97]. The scarcity of such records could be somewhat alleviated not only by dedicating more effort to the recovery of associated parasites but also by implementing less aggressive preparation techniques on potential host specimens [96,97]. Ultimately, the results of this study may serve as a source of inspiration for the development and fine-tuning of biomimetic surfaces that involve simple denticulated designs for antifouling purposes [98–102].

## Data Availability

Data and R code are available from the Figshare database: (doi:10.6084/m9.figshare.18014312). The data are provided in electronic supplementary material [103].

## References

[1] Jacoby DM, Croft DP, Sims DW. 2012 Social behaviour in sharks and rays: analysis, patterns and implications for conservation. Fish Fish. **13**, 399-417. (10.1111/j.1467-2979.2011.00436.x)

[2] Magnhagen C, Braithwaite V, Forsgren E, Kapoor BG. 2008 Fish behaviour, 1st edn. London, UK: Science Publishers.

[3] Paton-Dominguez D. 2019 Cetaceans : evolution, behavior and conservation. New York, NY: Nova Science Publishers Inc.

[4] Dobson AP, Poole J. 1998 Conspecific aggregation and conservation biology. In Behavioral ecology and conservation biology (ed. T Caro), pp. 193-208. Oxford, UK: Oxford University Press.

[5] Marras S, Killen SS, Lindström J, McKenzie DJ, Steffensen JF, Domenici P. 2015 Fish swimming in schools save energy regardless of their spatial position. Behav. Ecol. Sociobiol. **69**, 219-226. (10.1007/s00265-014-1834-4) 25620833 PMC4293471

[6] Krause J, Ruxton GD. 2002 Living in groups. Oxford, UK: Oxford University Press.

[7] Hoare DJ, Couzin ID, Godin J-G, Krause J. 2004 Context-dependent group size choice in fish. Anim. Behav. **67**, 155-164. (10.1016/j.anbehav.2003.04.004)

[8] Domenici P. 2001 The scaling of locomotor performance in predator–prey encounters: from fish to killer whales. Comp. Biochem. Physiol. Part A Mol. Integr. Physiol. **131**, 169-182. (10.1016/S1095-6433(01)00465-2) 11733175

[9] Johnson DD, Kays R, Blackwell PG, Macdonald DW. 2002 Does the resource dispersion hypothesis explain group living? Trends Ecol. Evol. **17**, 563-570. (10.1016/S0169-5347(02)02619-8)

[10] Barber I, Hoare D, Krause J. 2000 Effects of parasites on fish behaviour: a review and evolutionary perspective. Rev. Fish Biol. Fish. **10**, 131-165. (10.1023/A:1016658224470)

[11] Hudson PJ, Rizzoli AP, Grenfell BT, Heesterbeek JAP, Dobson AP. 2002 Ecology of wildlife diseases. Oxford, UK: Oxford University Press.

[12] Richards EL, Van Oosterhout C, Cable J. 2010 Sex-specific differences in shoaling affect parasite transmission in guppies. PLoS ONE **5**, e13285. (10.1371/journal.pone.0013285) 20949014 PMC2952601

[13] Sasal P. 2003 Experimental test of the influence of the size of shoals and density of fish on parasite infections. Coral Reefs **22**, 241-246. (10.1007/s00338-003-0313-6)

[14] Dobson AP. 1988 The population biology of parasite-induced changes in host behavior. Q. Rev. Biol. **63**, 139-165. (10.1086/415837) 3045862

[15] Poulin R, Morand S. 2000 The diversity of parasites. Q. Rev. Biol. **75**, 277-293. (10.1086/393500) 11008700

[16] Seilacher A, Reif W-E, Wenk P. 2007 The parasite connection in ecosystems and macroevolution. Naturwissenschaften **94**, 155-169. (10.1007/s00114-006-0164-4) 17111182

[17] Dawkins R, Krebs JR. 1979 Arms races between and within species. Proc. R. Soc. B **205**, 489-511. (10.1098/rspb.1979.0081) 42057

[18] Buonocore F, Gerdol M. 2016 Alternative adaptive immunity strategies: coelacanth, cod and shark immunity. Mol. Immunol. **69**, 157-169. (10.1016/j.molimm.2015.09.003) 26423359

[19] Behringer DC, Karvonen A, Bojko J. 2018 Parasite avoidance behaviours in aquatic environments. Phil. Trans. R. Soc. B **373**, 20170202. (10.1098/rstb.2017.0202) 29866915 PMC6000143

[20] Wisenden BD, Goater CP, James CT. 2019 Behavioral defenses against parasites and pathogens. In Fish defenses (eds G Zaccone, C Perrière, A Mathis, BG Kapoor), pp. 151-168. London, UK: Science Publishers. (10.1201/9780429187285-5)

[21] Raschi WG, Tabit C. 1992 Functional aspects of placoid scales: a review and update. Mar. Freshw. Res. **43**, 123-147. (10.1071/MF9920123)

[22] Pollerspöck J, Straube N. 2015 Bibliography database of living/fossil sharks, rays and chimaeras (chondrichthyes: elasmobranchii, holocephali) -host-parasites list/parasite-hosts list-. 2nd edn. World Wide Web electronic publication; see https://shark‐references.com/.

[23] Benz GW, Bullard SA. 2004 Metazoan parasites and associates of chondrichthyans with emphasis on taxa harmful to captive hosts. In The elamobranch husbandry manual: captive care of sharks, rays, and their relatives (eds M Smith, D Warmolts, D Thoney, R Hueter), pp. 325-416. Columbus, OH: Ohio Biological Survey.

[24] Woo PT, Buchmann K. 2012 Fish parasites: pathobiology and protection. Wallingford, UK: CABI.

[25] Meyer W, Seegers U. 2012 Basics of skin structure and function in elasmobranchs: a review. J. Fish Biol. **80**, 1940-1967. (10.1111/j.1095-8649.2011.03207.x) 22497413

[26] Reif W-E. 1985 Squamation and ecology of sharks. Frankfurt am Main, Germany: Courier Forschungsinstitut Senckenberg.

[27] Ferrón HG, Botella H. 2017 Squamation and ecology of thelodonts. PLoS ONE **12**, e0172781. (10.1371/journal.pone.0172781) 28241029 PMC5328365

[28] Reif W-E. 1982 Morphogenesis and function of the squamation in sharks. Comparative functional morphology of shark scales, and ecology of sharks. Neues Jahrb. fur Geol. Palaontol. - Abh. **164**, 172-183. (10.1127/njgpa/164/1982/172)

[29] Reif W-E, Dinkelacker A. 1982 Hydrodynamics of the squamation in fast swimming sharks. Neues Jahrb. fur Geol. Palaontol. - Abh. **164**, 184-187. (10.1127/njgpa/164/1982/184)

[30] Bechert D, Reif W-E. 1985 On the drag reduction of the shark skin. In 23rd aerospace sciences meeting, Reno, NV, 14–17 January 1985, pp. 546. Colorado, USA: American Institute of Aeronautics and Astronautics. (10.2514/6.1985-546)

[31] Raschi WG, Musick JA. 1984 Hydrodynamic aspects of shark scales. Special report in applied marine science and ocean engineering, no. 272, pp. 1–82. (10.21220/V5TQ6B)

[32] Reif W-E. 1978 Protective and hydrodynamic function of the dermal skeleton of elasmobranchs. Neues Jahrb. fur Geol. Palaontol. - Abh. **157**, 133-141.

[33] Reif W-E. 1985 Functions of scales and photophores in mesopelagic luminescent sharks. Acta Zool. **66**, 111-118. (10.1111/j.1463-6395.1985.tb00829.x)

[34] Ferrón HG, Paredes-Aliaga MV, Martínez-Pérez C, Botella H. 2018 Bioluminescent-like squamation in the galeomorph shark *Apristurus ampliceps* (Chondrichthyes: Elasmobranchii). Contrib. Zool. **87**, 187-196. (10.1163/18759866-08703004)

[35] Grover CA. 1974 Juvenile denticles of the swell shark *Cephaloscyllium ventriosum*: function in hatching. Can. J. Zool. **52**, 359-363. (10.1139/z74-043)

[36] Southall EJ, Sims DW. 2003 Shark skin: a function in feeding. Proc. R. Soc. B **270**, 47-49. (10.1098/rsbl.2003.0006) PMC1698022 12952633

[37] Dean B, Bhushan B. 2010 Shark-skin surfaces for fluid-drag reduction in turbulent flow: a review. Phil. Trans. R. Soc. A **368**, 4775-4806. (10.1098/rsta.2010.0201) 20855320

[38] Oeffner J, Lauder GV. 2012 The hydrodynamic function of shark skin and two biomimetic applications. J. Exp. Biol. **215**, 785-795. (10.1242/jeb.063040) 22323201

[39] Crooks N, Waring CP. 2013 Sexual dimorphisms in the dermal structure of the lesser-spotted catshark, *Scyliorhinus canicula* (Linnaeus, 1758). Acta Zool. **94**, 331-334. (10.1111/j.1463-6395.2012.00560.x) PMC3792157 24116179

[40] Crooks N, Babey L, Haddon WJ, Love AC, Waring CP. 2013 Sexual dimorphisms in the dermal denticles of the lesser-spotted catshark, *Scyliorhinus canicula* (Linnaeus, 1758). PLoS ONE **8**, e76887. (10.1371/journal.pone.0076887) 24116179 PMC3792157

[41] Straube N, Li C, Claes JM, Corrigan S, Naylor GJ. 2015 Molecular phylogeny of Squaliformes and first occurrence of bioluminescence in sharks. BMC Evol. Biol. **15**, 162. (10.1186/s12862-015-0446-6) 26277575 PMC4537554

[42] Benz GW, Borucinska JD, Lowry LF, Whiteley HE. 2002 Ocular lesions associated with attachment of the copepod *Ommatokoita elongata* (Lernaeopodidae: Siphonostomatoida) to corneas of Pacific sleeper sharks *Somniosus pacificus* captured off Alaska in Prince William Sound. J. Parasitol. **88**, 474-481. (10.1645/0022-3395(2002)088[0474:OLAWAO]2.0.CO;2) 12099414

[43] Feld K, Kolborg AN, Nyborg CM, Salewski M, Steffensen JF, Berg-Sørensen K. 2019 Dermal denticles of three slowly swimming shark species: microscopy and flow visualization. Biomimetics **4**, 38. (10.3390/biomimetics4020038) 31137624 PMC6631580

[44] Ingram AL, Parker AR. 2006 The functional morphology and attachment mechanism of pandarid adhesion pads (Crustacea: Copepoda: Pandaridae). Zool. Anz. **244**, 209-221. (10.1016/j.jcz.2005.11.001)

[45] Pollerspöck J, Straube N. 2021 Bibliography Database of living/fossil sharks, rays and chimaeras (Chondrichthyes: Elasmobranchii, Holocephali). See www.shark-references.com.

[46] Ebert DA, Dando M, Fowler S. 2021 Sharks of the world: A complete guide. New Jersey, NJ: Princeton University Press.

[47] IUCN. 2021 The IUCN Red List of Threatened Species. Version 2021-2. See https://www.iucnredlist.org.

[48] Froese R, Pauly D. 2021 FishBase. See www.fishbase.org.

[49] Schneider CA, Rasband WS, Eliceiri KW. 2012 NIH Image to ImageJ: 25 years of image analysis. Nat. Methods **9**, 671-675. (10.1038/nmeth.2089) 22930834 PMC5554542

[50] Orme D, Freckleton R, Thomas G, Petzoldt T, Fritz S, Isaac N, Pearse W. 2013 *The caper package: comparative analysis of phylogenetics and evolution in R*. R package v. 1.0.1

[51] R Development Core Team. 2020 R: A language and environment for statistical computing. Vienna, Austria: R Foundation for Statistical Computing.

[52] Symonds MR, Blomberg SP. 2014 A primer on phylogenetic generalised least squares. In Modern phylogenetic comparative methods and their application in evolutionary biology (ed. L Garamszegi), pp. 105-130. New York, NY: Springer. (10.1007/978-3-662-43550-2_5)

[53] Bordes F, Morand S. 2009 Parasite diversity: an overlooked metric of parasite pressures? Oikos **118**, 801-806. (10.1111/j.1600-0706.2008.17169.x)

[54] Walther BA, Cotgreave P, Price RD, Gregory RD, Clayton DH. 1995 Sampling effort and parasite species richness. Parasitol. Today **11**, 306-310. (10.1016/0169-4758(95)80047-6) 15275331

[55] Lüdecke D, Makowski D, Ben-Shachar MS, Patil I, Waggoner P, Wiernik BM, Arel-Bundock V. 2022 *performance: Assessment of Regression Models Performance*. See https://CRAN.R-project.org/package=performance.

[56] Jackman S. 2020 *Political Science Computational Laboratory. R package v. 1.5.5*.

[57] Merkle E, You D, Schneider L, Bae S. 2020 *nonnest2: Tests of Non-Nested Models*. See https://CRAN.R-project.org/package=nonnest2.

[58] Vuong QH. 1989 Likelihood ratio tests for model selection and non-nested hypotheses. Econometrica **57**, 307-333. (10.2307/1912557)

[59] Ben-Shachar MS, Makowski D, Lüdecke D, Patil I, Wiernik BM, Kelley K, Stanley D, Burnett J, Karreth J. 2022 effectsize: Indices of Effect Size and Standardized Parameters. See https://CRAN.R-project.org/package=effectsize.

[60] Fox J, Weisberg S. 2018 An R companion to applied regression. Thousand Oaks, CA: SAGE Publications.

[61] James G, Witten D, Hastie T, Tibshirani R. 2017 An introduction to statistical learning: with applications in R. New York, NY: Springer.

[62] Vélez-Zuazo X, Agnarsson I. 2011 Shark tales: a molecular species-level phylogeny of sharks (Selachimorpha, Chondrichthyes). Mol. Phylogenet. Evol. **58**, 207-217. (10.1016/j.ympev.2010.11.018) 21129490

[63] Paradis E, Blomberg S, Bolker B, Brown J, Claude J, Cuong HS, Desper R, Didier G. 2019 *Package ‘ape’: Analyses of phylogenetics and evolution, R package v. 5.5*.

[64] Bush AO, Lafferty KD, Lotz JM, Shostak AW. 1997 Parasitology meets ecology on its own terms: Margolis *et al*. revisited. J. Parasitol. **83**, 575-583. (10.2307/3284227) 9267395

[65] Shaw AK, Sherman J, Barker FK, Zuk M. 2018 Metrics matter: the effect of parasite richness, intensity and prevalence on the evolution of host migration. Proc. R. Soc. B **285**, 20182147. (10.1098/rspb.2018.2147) PMC6253363 30429312

[66] Russo RA. 1975 Notes on external parasites of California inshore sharks. Calif. Fish Game. **61**, 228-232.

[67] Hine M, Jones JB. 2000 A checklist of the parasites of New Zealand fishes including previously unpublished records. NIWA Technical Report 75. Wellington, New Zealand: NIWA.

[68] Klimpel S, Palm HW, Seehagen A. 2003 Metazoan parasites and food composition of juvenile *Etmopterus spinax* (L., 1758) (Dalatiidae, Squaliformes) from the Norwegian Deep. Parasitol. Res. **89**, 245-251. (10.1007/s00436-002-0741-1) 12632160

[69] Orłowska K. 1979 Parasites of North Sea spiny dogfish, *Squalus acanthias* L.(Selachiiformes. Squalidae). Acta Ichthyol. Piscat. **9**, 33-44. (10.3750/AIP1979.09.1.03)

[70] Purivirojkul W, Chaidee P, Thapanand-Chaidee T. 2009 Parasites of deep-sea sharks from the Andaman Sea with six new records of parasites in Thailand. Kasetsart J. (Nat. Sci.) **43**, 93-99.

[71] Wierzbicka J, Langowska D. 1984 Parasitic fauna of spiny dogfish *Squalus acanthias* L. off New Zealand. Acta Ichthyol. Piscat. **14**, 157-166. (10.3750/aip1984.14.1-2.11)

[72] Cressey RF. 1967 Caligoid copepods parasitic on sharks in the Indian Ocean. Proc. Natl Acad. Sci. USA **121**, 1-21. (10.5479/si.00963801.121-3572.1)

[73] Newbound DR, Knott B. 1999 Parasitic copepods from pelagic sharks in western Australia. Bull. Mar. Sci. **65**, 715-724.

[74] Benz GW. 1989 Developmental stages of Alebion lobatus Cressey, 1970 (Copepoda: Euryphoridae) found parasitic on the sandbar shark (*Carcharhinus plumbeus* (Nardo, 1827)) in the western North Atlantic, and a phylogenetic analysis of the genus *Alebion* *Krøyer*, 1863. Can. J. Zool. **67**, 1578-1598. (10.1139/z89-224)

[75] Kearn GC. 1965 The biology of *Leptocotyle minor*, a skin parasite of the dogfish, *Scyliorhinus canicula*. Parasitology **55**, 473-480. (10.1017/S0031182000069183)

[76] Grutter AS. 2002 Cleaning symbioses from the parasites’ perspective. Parasitology **124**, 65-81. (10.1017/S0031182002001488) 12396217

[77] Fletcher T, Altringham J, Peakall J, Wignall P, Dorrell R. 2014 Hydrodynamics of fossil fishes. Proc. R. Soc. B **281**, 20140703. (10.1098/rspb.2014.0703) PMC4083790 24943377

[78] Claes JM, Mallefet J. 2009 Bioluminescence of sharks: first synthesis. In Bioluminescence in focus – A collection of illuminating essays (ed. VB Meyer-Rochow), pp. 51-65. Kerala, India: Research Signpost.

[79] Duchatelet L, Claes JM, Delroisse J, Flammang P, Mallefet J. 2021 Glow on sharks: state of the art on bioluminescence research. Oceans **2**, 822-842. (10.3390/oceans2040047)

[80] Duchatelet L, Marion R, Mallefet J. 2021 A third luminous shark family: confirmation of luminescence ability for *Zameus squamulosus* (Squaliformes; Somniosidae). Photochem. Photobiol. **97**, 739-744. (10.1111/php.13393) 33529364

[81] Shoval O, Sheftel H, Shinar G, Hart Y, Ramote O, Mayo A, Dekel E, Kavanagh K, Alon U. 2012 Evolutionary trade-offs, Pareto optimality, and the geometry of phenotype space. Science **336**, 1157-1160. (10.1126/science.1217405) 22539553

[82] Tendler A, Mayo A, Alon U. 2015 Evolutionary tradeoffs, Pareto optimality and the morphology of ammonite shells. BMC Syst. Biol. **9**, 12. (10.1186/s12918-015-0149-z) 25884468 PMC4404009

[83] Bowman TE. 1971 *Palaega lamnae*, new species (Crustacea; isopoda) from the upper Cretaceous of Texas. J. Paleontol. **45**, 540-541.

[84] Cressey R, Patterson C. 1973 Fossil parasitic copepods from a lower cretaceous fish. Science **180**, 1283-1285. (10.1126/science.180.4092.1283) 17759124

[85] Cressey R, Boxshall G. 1989 *Kabatarina pattersoni*, a fossil parasitic copepod (Dichelesthiidae) from a Lower Cretaceous fish. Micropaleontology **35**, 150-167. (10.2307/1485466)

[86] Upeniece I. 1996 *Lodeacanthus gaujicus* n. g. et sp. (Acanthodii: Mesacanthidae) from the Late Devonian of Latvia. Mod. Geol. **20**, 383-398.

[87] Upeniece I. 2001 The unique fossil assemblage from the Lode Quarry (Upper Devonian, Latvia). Foss. Rec. **4**, 101-119. (10.1002/mmng.20010040108)

[88] Nagler C, Haug C, Resch U, Haug JT. 2016 150 million years old isopods on fishes: a possible case of palaeo-parasitism. Bull. Geosci. **91**, 1-12. (10.3140/bull.geosci.1586)

[89] Stinnesbeck ES, Wägele JW, Herder F, Rust J, Stinnesbeck W. 2022 A fish-parasitic isopod (Cymothoidae) on the pachyrhizodont Goulmimichthys roberti from the lower Turonian (Upper Cretaceous) Vallecillo plattenkalk, NE Mexico. Cretac. Res. **129**, 105019. (10.1016/j.cretres.2021.105019)

[90] Petit G. 2010 Skin nodules in fossil fishes from Monte Bolca (Eocene. Northern Italy). Geodiversitas **32**, 157-163. (10.5252/g2010n1a5)

[91] Petit G, Khalloufi B. 2012 Paleopathology of a fossil fish from the Solnhofen Lagerstätte (Upper Jurassic, southern Germany). Int. J. Paleopathol. **2**, 42-44. (10.1016/j.ijpp.2012.07.001) 29539352

[92] De Baets K, Dentzien-Dias P, Upeniece I, Verneau O, Donoghue PCJ. 2015 Chapter Three - Constraining the deep origin of parasitic flatworms and host-interactions with fossil evidence. In Advances in parasitology (eds K De Baets, DTJ Littlewood), pp. 93-135. San Diego, CA: Academic Press. 10.1016/bs.apar.2015.06.002 26597066

[93] Leung TLF. 2017 Fossils of parasites: what can the fossil record tell us about the evolution of parasitism? Biol. Rev. **92**, 410-430. (10.1111/brv.12238) 26538112

[94] De Baets K, Huntley JW. 2021 The evolution and fossil record of parasitism: identification and macroevolution of Parasites. Berlin, Germany: Springer Nature.

[95] De Baets K, Huntley JW, Scarponi D, Klompmaker AA, Skawina A. 2021 Phanerozoic parasitism and marine metazoan diversity: dilution versus amplification. Phil. Trans. R. Soc. B **376**, 20200366. (10.1098/rstb.2020.0366) 34538136 PMC8450635

[96] De Baets K, Huntley JW, Klompmaker AA, Schiffbauer JD, Muscente AD. 2021 The fossil record of parasitism: its extent and taphonomic constraints. In The evolution and fossil record of parasitism: coevolution and paleoparasitological techniques. Topics in geobiology (eds K De Baets, JW Huntley), pp. 1-50. Berlin, Germany: Springer.

[97] Leung TLF. 2021 Parasites of fossil vertebrates: what we know and what can we expect from the fossil record? In The evolution and fossil record of parasitism: identification and macroevolution of parasites (eds K De Baets, JW Huntley), pp. 1-27. Cham, Switzerland: Springer International Publishing.

[98] Chien H-W, Chen X-Y, Tsai W-P, Lee M. 2020 Inhibition of biofilm formation by rough shark skin-patterned surfaces. Colloids Surf. B **186**, 110738. (10.1016/j.colsurfb.2019.110738) 31869602

[99] Ibrahim MD, Philip S, Lam SS, Sunami Y. 2021 Evaluation of an antifouling surface inspired by Malaysian Sharks *Negaprion Brevirostris* and *Carcharhinus Leucas* Riblets. Tribol. Int. **16**, 70-80. (10.2474/trol.16.70)

[100] Munther M, Palma T, Angeron IA, Salari S, Ghassemi H, Vasefi M, Beheshti A, Davami K. 2018 Microfabricated biomimetic placoid scale-inspired surfaces for antifouling applications. Appl. Surf. Sci. **453**, 166-172. (10.1016/j.apsusc.2018.05.030)

[101] Peng YL, Lin CG, Wang L. 2009 The preliminary study on antifouling mechanism of shark skin. Adv. Mater. Res. **79–82**, 977-980. (10.4028/www.scientific.net/AMR.79-82.977)

[102] Rostami S, Garipcan B. 2020 Evolution of antibacterial and antibiofouling properties of sharkskin-patterned surfaces. Surf. Innov. **10**,165-190. (10.1680/jsuin.21.00055)

[103] Ferrón HG, Palacios-Abella JF. 2022 Grouping behaviour impacts on the parasitic pressure and squamation of sharks. FigShare. (10.6084/m9.figshare.c.5965240) PMC9115038 35582806

